# Monotropein Protects against Inflammatory Bone Loss and Suppresses Osteoclast Formation and Bone Resorption by Inhibiting NFATc1 via NF-κB and Akt/GSK-3β Pathway

**DOI:** 10.3390/nu14193978

**Published:** 2022-09-24

**Authors:** Qi Zhang, Sijing Hu, Yuqiong He, Zile Song, Yi Shen, Zihui Zhao, Quanlong Zhang, Luping Qin, Qiaoyan Zhang

**Affiliations:** 1 School of Pharmaceutical Sciences, Zhejiang Chinese Medical University, Hangzhou 310053, China; 2 Institute of Chinese Materia Madica, Shanghai University of Traditional Chinese Medicine, Shanghai 201203, China

**Keywords:** monotropein, inflammatory bone loss, osteoclast, NFATc1, Akt/GSK3β pathway

## Abstract

Monotropein (Mon) is a kind of iridoid glycoside plant secondary metabolite primarily present in some edible and medicinal plants. The aim of this study was to investigate the effect of Mon on lipopolysaccharide (LPS)-induced inflammatory bone loss in mice and osteoclasts (OCs) derived from bone marrow-derived macrophages (BMMs), and explore the mechanisms underlying the effect of Mon on LPS-induced osteoclastogenesis. It was found that Mon markedly attenuated deterioration of the bone micro-architecture, enhanced tissue mineral content (TMC) and bone volume/total volume (BV/TV), reduced structure model index (SMI) and trabecular separation/spacing (Tb.Sp) in the bone tissue and decreased the activities of tartrate resistant acid phosphatase-5b (TRACP-5b), receptor activator NF-κB (RANK), and receptor activator NF-κB ligand (RANKL) as well as the serum levels of interleukin 6 (IL-6) and interleukin 1β (IL-1β) in LPS-treated mice. In addition, Mon treatment reduced the number of TRAP positive OCs in the bone tissue of LPS-treated mice and also exerted a stronger inhibitory effect on formation, differentiation, and F-actin ring construction of OCs derived from BMMs. Mon significantly inhibited the expression of the nuclear factor of activated T-cells c1 (NFATc1) and the immediate early gene (C-Fos) and nuclear translocation of NFATc1 in LPS-treated OCs, thereby inhibiting the expression of matrix metalloproteinase-9 (MMP-9), cathepsin K (CtsK), and TRAP. Mon significantly inhibited the expression of TRAF6, phosphorylation of P65, and degradation of IKBα, thus inhibiting the activation of NF-κB pathway in LPS-induced inflammatory mice and OCs derived from BMMs, and also inhibited LPS-induced phosphorylation of protein kinase B (Akt) and Glycogen synthase kinase 3β (GSK-3β) in OCs derived from BMMs. In conclusion, these results suggested that Mon could effectively inhibit osteoclastogenesis both in vitro and in vivo and therefore may prove to be potential option for prevention and treatment of osteoclastic bone resorption-related diseases.

## 1. Introduction

Bone remodeling is an important process to maintain bone development and growth, involving the coupling of bone-forming osteoblasts (OBs) and bone-resorbing osteoclasts (OCs). Homeostatic balance of bone remodeling is required for the maintenance of healthy bones [1]. Inflammation has been demonstrated to cause imbalance of bone remodeling, leading to bone loss. Some inflammatory diseases such as systemic lupus erythematous (SLE), rheumatoid arthritis (RA), cystic fibrosis (CF), chronic obstructive pulmonary disease (COPD), inflammatory bowel disease, and periodontal disease, have a great impact on bone health by causing systemic and/or local bone loss. In addition, many inflammatory mediators such as interleukin (IL)-1, IL-6, IL-17, and tumor necrosis factor (TNF) have been implicated in disturbing bone homeostasis by decreasing bone formation and increasing bone resorption. Therefore, patients with inflammatory diseases are at a significantly higher risk of developing skeletal diseases such as osteoporosis or osteopenia [2,3,4]. Given the importance of inflammation in driving destructive bone remodeling, it is urgent to develop potential therapeutic medications for inflammatory disease-associated bone loss.

OCs are unique effector cells for bone resorption. Over-activation of OCs by inflammatory response expedites perturbation of steady-state bone remodeling, leading to excess bone resorption and then bone destruction and impaired bone quality [5]. OCs are multinucleated cells derived from bone marrow-derived macrophages (BMMs). Macrophage colony stimulating factor (M-CSF) and receptor activator of NF-κB ligand (RANKL) are required for the differentiation of BMMs into OCs. RANKL binds with its receptors RANK activate various downstream signaling, such as nuclear factor kappa B (NF-κB), extracellular regulated protein kinase (ERK), c-Jun N terminal protein kinase (JNK), p38, and the phosphatidylinositol 3-kinase (PI3K)/akt serine/threonine kinase (Akt) pathway, and subsequently regulates nuclear factor of activated T-cells c1 (NFATc1) to induce formation and differentiation of OCs [6,7,8]. Furthermore, NFATc1 also activates the expression of c-Fos, contributing to the increased expression of OC-specific genes such as tartrate-resistant acid phosphatase (TRAP), MMP-9 and cathepsin K (CTSK), which stimulates OC activity, resulting in bone resorption [9].

NF-κB pathway activation in inflammation induces the expression of NFATc1. Glycogen synthase kinase-3β (GSK-3β) is a negative regulator of NFATc1, and GSK-3β activity is inhibited by Akt-dependent phosphorylation. Therefore, NF-κB and the Akt/GSK-3β signaling cascade are critical during RANKL-induced OC differentiation [10]. Lipopolysaccharide (LPS), as an important pathogen in inflammatory disease, can induce the production of various cytokines and mediators in macrophages such as TNF-α, IL-1β, and PGE2 and then initiate the expression of RANKL and NFATc1, thus facilitating the formation, fusion, and survival of OCs [11,12].

Monotropein (Mon) is a kind of iridoid glycoside plant secondary metabolite that primarily occurs in some edible and medicinal plants, such as *Morind officinalis* HOW., *Pyrola decorate*, *Gaultheria phillyreifolia* (berries), *Gaultheria poeppigii*, *Morinda citrifolia* L., *Vaccinium macrocarpon* (American cranberry), *Vaccinium oxycoccus* (small cranberry), *Vaccinium vitis-idaea* (lingonberry), and *Vaccinium myrtillus* (bilberry) [13]. It is reported that Mon possesses a variety of pharmacological activities, including anti-osteoporosis, anti-osteoarthritis, anti-arthritic, antioxidant, anti-inflammatory and anti-apoptotic effects [14]. Our previous study demonstrated that Mon prevented bone loss induced by combination treatment of LPS and ovariectomy in mice and enhanced the bone forming activities of MC3T3-E1 cells injured by LPS through decreasing the production of inflammatory cytokines via inhibiting activation of the NF-κB pathway [15]. In the present study, we aimed to investigate the inhibitory effect of Mon on osteoclastic bone resorption in LPS-stimulated mice and LPS-induced OCs derived from BMMs, and its regulatory effect on NF-κB and Akt/GSK-3β pathways in OCs, explore the underlying mechanism of Mon in inhibiting bone resorption, hoping that the obtained results could provide a scientific basis for the application of edible and medicinal plants containing this compound in the prevention and treatment of inflammatory bone diseases.

## 2. Materials and Methods

### 2.1. Chemicals and Reagents

Chemicals and reagents Mon used in this study were obtained from Yuan ye Biology Technology (Shanghai, China); fetal bovine serum (FBS); α-modified minimal essential medium (α-MEM); and penicillin/streptomycin were purchased from Gibco BRL(Grand Island, NY, USA); receptor activator of nuclear factor kB ligand (RANKL); and macrophage colony-stimulating factor (M-CSF) were purchased from PeproTech EC Ltd. (London, UK); BCA protein assay kit were purchased from Beyotime Biotechnology Inc.(Shanghai, China); antibodies against IkBα, c-Fos, MMP9, AKT, P-AKT, and TRAF6 were obtained from Boster Biological Technology (Wuhan, China); antibodies against GSK-3β, P- GSK-3β, p65, p-p65, NFATc1, and GAPDH were purchased from Cell Signaling Technology Inc.(Beverly, MA, USA). and antibody against CtsK were purchased from Abcam. (Cambridge, UK).

### 2.2. Animal Experimental Protocol

#### 2.2.1. Animals and Diet

The LPS-induced bone destruction model was constructed in 40 C57/BL6 female mice aged 8 weeks and the mice were provided by SLAC Laboratory Animal Company. (Shanghai, China) (License No. SCXK (Hu) 2017-0005). The animals were maintained at the Experimental Animal Center of Zhejiang Chinese Medical University (Hangzhou, China) under controlled conditions of 22–24 °C, 50–60% humidity with a 12 h light/dark cycle, with free access to water and food. The experimental protocol was approved by the Experimental Animal Ethics Committee of the said university. Forty healthy C57/BL6 mice were equally randomized to four groups: PBS control group, 5 mg/kg LPS treatment group, combination treatment group using 5 mg/kg LPS and 40 mg/kg Mon, and combination treatment group using 5 mg/kg LPS and 80 mg/kg Mon. Mice were injected intraperitoneally with LPS or the same volume of PBS on day 2 and 6, and Mon was given at day 1, 3, 5, 7 and 9 [16]. At day 10, the mice were fasted for 12 h, and urine was collected from each mouse. Then, all mice were sacrificed, and the blood was collected from the carotid artery and centrifuged at 3000 rpm for 10 min to collect sera, which were then stored at −20 °C prior to biomarker assay, while the right femurs were collected and fixed in 4% formaldehyde for Micro-CT scanning.

#### 2.2.2. Measurement of Biomarker Levels in Urine and Serum

Serum levels of biomarkers including PICP, TRACP-5b, RANK, sRANKL, IL-1β, and IL-6 were detected using ELISA kits according to the manufacturer’s instructions. The absorbance was measured with a microplate reader (Thermo Fisher Scientific Inc., Pittsburgh, PA, USA).

#### 2.2.3. Micro-CT Analysis

The left posterior femur was scanned by Micro-CT (GE Healthcare company, boston, MA, USA) using the following instrument parameters: 80 kV, 80 μA, 0.4° rotation step, and trabecular bone parameters were measured, including bone mineral density (BMD) and bone morphometric parameters.

#### 2.2.4. HE and TRAP Staining

The right posterior femur was decalcified at room temperature in 10% EDTA for 14 days, paraffin-embedded, sliced into sections, and stained with HE or TRAP to observe alterations in bone histological structures and OC activities.

#### 2.2.5. Western Blot

After removing the excess muscle tissue and washing off the bone marrow with PBS, the right anterior femur was treated with the IP lysate solution containing protease and phosphatase inhibitor, and then ground in a tissue homogenizer for 20 min. The protein content was determined using a BCA protein quantification kit (Beyotime Biotechnology Inc., Shanghai, China) following the manufacturer’s protocol. Total proteins were transferred onto a polyvinylidene difluoride membrane, which was blocked in non-fat milk for 2 h at room temperature and incubated overnight with the specific primary antibodies at 4 °C, using GAPDH as the protein internal standard and the standard for quantifying protein expression. The specific primary antibodies used were IkBα, c-Fos, MMP9, AKT, p-AKT, TRAF6, GSK-3β, p-GSK-3β, p65, p-p65, and NFATc1.

### 2.3. Cell Experiment Protocol

#### 2.3.1. Cell Culture

Bone marrow macrophages were isolated from the femurs of 6-week-old C57BL/6 mice and cultured in a dish containing α-MEM supplemented with 10% FBS, 100 U/mL penicillin, 100 mg/mL streptomycin, and 5 ng/mL M-CSF in humidified atmosphere of 5% CO_2_ for 24 h. Non-adherent cells were cultured in a complete medium containing 30 ng/mL M-CSF for 3 days to obtain bone marrow macrophages (BMMs).

BMMs were seeded into 96-well plates at a density of 1 × 10^4^/well, allowed to adhere overnight, and then treated with Mon at concentrations of 0.1, 1, and 10 μM in the presence of 30 ng/mL M-CSF for 48 h. Cell proliferation was measured by CCK-8 assay. Briefly, 100 μL complete medium and 10 μL CCK-8 solution were added into the culture well and incubated for appropriate time. The absorbance value was measured at 450 nm with a microplate reader (Thermo Fisher Scientific Inc., Pittsburgh, PA, USA).

#### 2.3.2. Determination of the Activity and Staining of OC TRAP

BMMs were seeded into 96-well plates at a density of 1 × 10^4^ cells/well and then treated with different concentrations of Mon (0.1, 1 and 10 μM) in the presence of 30 ng/mL M-CSF or 200 ng/mL LPS for 6 days. Cells were fixed with 4% paraformaldehyde and stained using a TRAP kit (Sigma, St Louis, MO, USA) following the manufacturer’s protocol. TRAP-positive cells containing three or more nuclei were counted as OCs. For TRAP activity, cells were treated with 20 µL 1% Triton X-100 at room temperature for 30 min, added with TRAP reaction, and then incubated at 37 °C for 30 min. The reaction was terminated by adding 100 µL 1 M NaOH. The absorbance was measured at 450 nm using a microplate reader. TRAP activity was expressed as the number of moles of p-nitrophenol produced per 100 OCs.

#### 2.3.3. Observation of F-Actin Rings of OCs

BMMs were treated as described in Section 2.3.3, fixed with 4% paraformaldehyde, and incubated in 0.1% Triton-PBS for 15 min to permeabilize the membrane. F-actin rings in OCs were stained with FITC-labeled phalloidin (Sigma, St. Louis, MO, USA) for 45 min. After washing, the glass coverslips were infiltrated with mounting medium containing DAPI (nuclear dye; Beyotime Biotechnology Inc., Shanghai, China), The F-actin rings of OCs were captured under a Carl Zeiss confocal laser scanning microscope (LSM880, Carl Zeiss, Oberkochen, Germany).

#### 2.3.4. Protein Extraction and Western Blot Analysis

BMMs were seeded into 6-well plates at a density of 1 × 10^5^ cells/well, stimulated with 30 ng/mL M-CSF and 20 ng/mL RANKL for 48 h, and then treated with different concentrations of Mon (0.1, 1 and 10 μM) for 4 h. Subsequently, 200 ng/mL LPS was added at the designated time points. Cells were washed twice with ice-cold PBS and then lysed using IP lysate containing protease and phosphatase inhibitor, using GAPDH as the protein internal standard and the standard for quantifying protein expression. The specific primary antibodies used were IkBa, c-Fos, MMP9, AKT, p-AKT, TRAF6, GSK-3β, p-GSK-3β, p65, p-p65, NFATc1, and GAPDH. The gray values of the band were quantified with the software of Image J.

#### 2.3.5. Immunofluorescence (IF) Assay

BMMs were treated in confocal culture dishes as described in Section 2.3.3, IF stained, incubated with anti-P65 (1:200), or anti-NFATc1 (1:200) for 2 h, and treated with Goat anti-rabbit IgG (H + L) highly cross-adsorbs secondary antibody (Thermo Fisher Scientific Inc, Pittsburgh, PA, USA) for 1 h. Images were captured under a Carl Zeiss confocal laser scanning microscope (LSM880, Carl Zeiss, Oberkochen, Germany).

### 2.4. Molecular Docking

The structure of AKT was prepared by the Protein Preparation Wizard Workflow provided in the Maestro module of Schrödinger software. The PDB ID of AKT is 6S9X. The protein was processed through the default pipeline. Subsequently, the Glidedock module of Schrödinger software was used to generate the protein grid of these complexes, and the grid boxes were defined as 12 × 12 × 12 Å, and the spatial region was centered on the original ligand of the complex structures. The 3D structure of the Mon compound was constructed by Chem3D Ultra 8.0; stereoisomers and tautomers were generated by LigPrep, and the protonation states of ligands at pH 7.0 ± 2.0 were predicted by Epik. Other parameters were set as default. Ligand docking module in Schrödinger was used to choose the receptor grid file and ligand file with extra-precision (XP) mode, which was generated from ligand preparation and protein preparation. The binding mode was visualized by Pymol [17].

### 2.5. Statistical Analysis

The Graphpad Prism 5.0 statistical software was used to analyze the experimental data. The analysis of ANOVA and Student’s *t*-test were applied to determine significant differences in experimental group data. Data are expressed as the mean ± standard deviation (SD) and the value of *p* < 0.05 was considered statistically significant.

## 3. Results

### 3.1. Mon Improves Micro-Architecture and Prevents Bone Loss in LPS-Treated Mice

**Figure 1:**
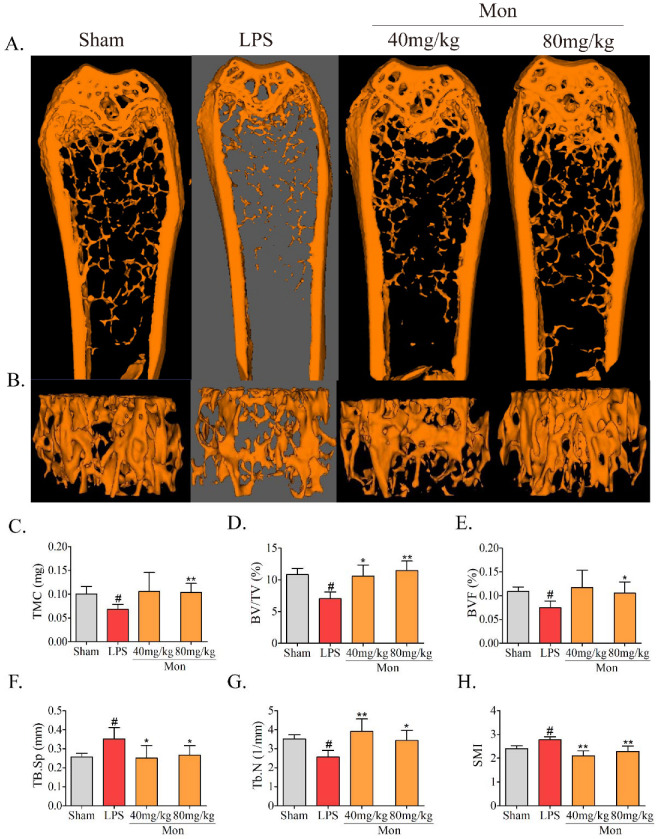
Mon improves the micro-architecture and prevents bone loss in LPS-treated mice (n = 8). (**A**,**B**) Representative images of micro-CT 2D and 3D microarchitecture of the femur. (**C**–**H**) Quantification of bone microarchitecture parameters, including TMC, BV/TV, BVF, Tb.Sp, Tb.N, and (**H**) SMI, respectively. Data are presented as the mean ± SD. # *p* < 0.05 compared with Sham group; * *p* < 0.05, ** *p* < 0.01 compared with LPS group.

As shown in Figure 1, treatment with LPS caused the deterioration of the micro-architecture of the bone tissue, as represented by thinner, less dense, and reduced connectivity and sparser trabeculae in the mouse femur, and the administration of Mon attenuated the deterioration of the bone micro-architecture in LPS treated mice. In addition, Micro-CT analysis showed that LPS treatment caused a significant decrease in tissue mineral content (TMC), mineralized bone volume to total volume (BV/TV) and trabecular number (Tb.N), and an increase in structure model index (SMI) and trabecular space (Tb.Sp), and Mon administration enhanced TMC and BV/TV and reduced SMI and Tb. Sp in the bone tissue of LPS-treated mice. These results demonstrated that Mon reversed the abnormal bone morphological parameters of the femoral cancellous bone induced by LPS.

### 3.2. Mon Is Involved into the Regulation of Bone Metabolism in LPS-Treated Mice

**Figure 2:**
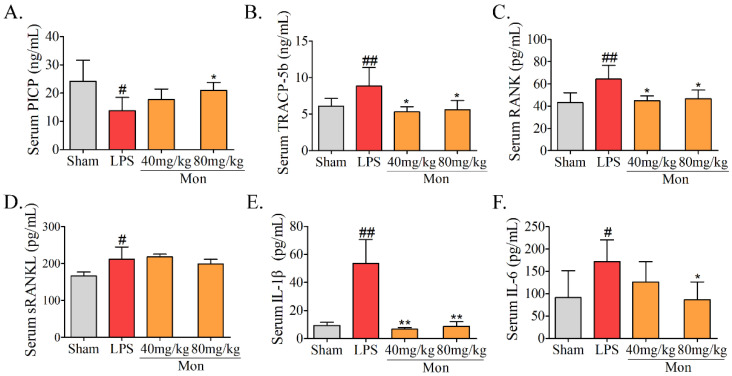
Mon is involved into the regulation of bone metabolism as assessed by level alterations of (**A**) PICP, (**B**) TRACP-5b, (**C**) RANK, (**D**) sRANKL, (**E**) IL-1β, and (**F**) IL-6 in serum of LPS-treated mice (n = 8). Data are presented as the mean ± SD. # *p* < 0.05, ## *p* < 0.01 compared with Sham group; * *p* < 0.05, ** *p* < 0.01 compared with LPS group.

The biochemical parameters are often used to evaluate the alteration of bone formation and bone resorption in animal experiments. As shown in Figure 2, LPS caused a significant decrease in PICP and a significant increase in TRAP-5b, RANK, and RANKL levels in the serum of mice. Mon significantly increased the level of PICP and decreased the activities of TRACP-5b, RANK, and RANKL in the serum of LPS-induced inflammatory mice. These results suggested that inflammation led to inhibition of bone formation and activation of bone resorption, and Mon treatment attenuated these effects of inflammation on bone metabolism.

LPS can activate the inflammatory responses, resulting in an increase in secretion of inflammatory cytokines. As shown in Figure 2, LPS treatment caused a significant increase in levels of IL-6 and IL-1β, while Mon significantly reduced the levels of IL-6 and IL-1β in serum of LPS-treated mice, indicating that Mon suppressed the activation of inflammation in LPS-treated mice.

### 3.3. Mon Reduces Osteoclastogenesis in Bone Tissue in LPS-Treated Mice and LPS-Treated BMMs

**Figure 3:**
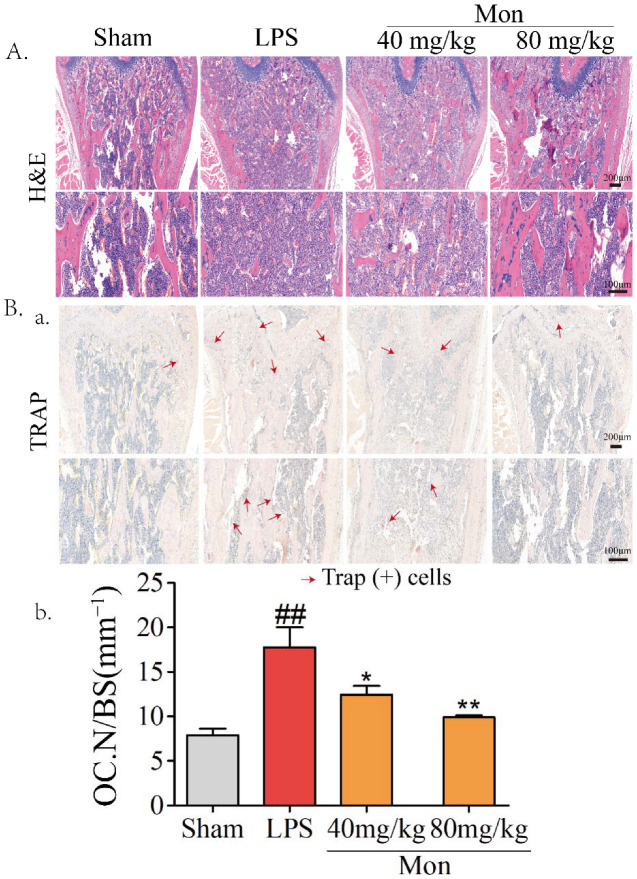
Mon attenuates the bone loss and decreases the number of osteoclasts in bone tissue of LPS-treated mice as evidenced by (**A**) HE and (**B**(**a**)) TRAP staining of the femora (n = 8). (**B**(**b**)) The quantifications of number of osteoclasts on the bone surface (Oc.N/BS) were calculated based on TRAP staining by using the Image J software. The data are expressed as means ± SD. ## *p* < 0.01 compared with Sham group; * *p* < 0.05, ** *p* < 0.01 compared with LPS group.

**Figure 4:**
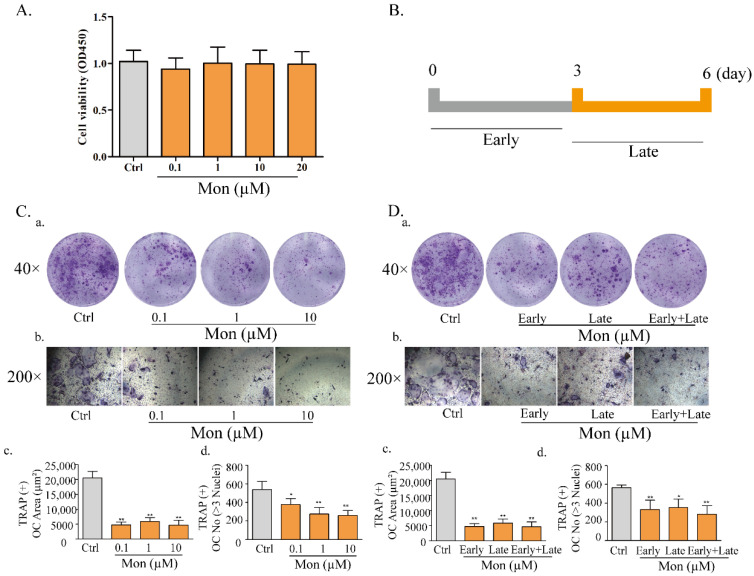
Mon reduces osteoclastogenesis in LPS-treated BMMs (n = 6). (**A**) The proliferation of osteoclast. (**C**) LPS−induced differentiation of BMMs into osteoclasts. BMMs were incubated with M-CSF (30 ng/mL), RANKL (20 ng/mL) for 48 h and then treated with different concentrations of Mon (0.1, 1, and 10 µM) in present of 30 ng/mL M-CSF, 200 ng/mL LPS for 6 days, then stained with Acid Phosphatase Kit. (**C**(**a**,**b**)) Morphologic characteristic of osteoclast stained for TRAP. The average area (μm^2^) (**C**(**c**)) and number (**C**(**d**)) of TRAP + OC with ≥3 nuclei. (**B**,**D**) The effects of Mon (10 µM) on formation of OC from BMMs during the 6-day process of stimulated by RANKL, LPS at different treatment times. (**D**(**a**,**b**)) Morphologic characteristic of osteoclast stained for TRAP. The average area (μm^2^) (**D**(**c**)) and number (**D**(**d**)) of TRAP + OC with ≥3 nuclei. The data are expressed as means ± SD. * *p* < 0.05, ** *p* < 0.01 compared with Ctrl group.

Our previous study reported the protective effect of Mon on impairment of inflammation on OB functions [15]. Considering the inhibitory effect of Mon on inflammation and bone resorption, we investigated the effect of Mon on osteoclastogenesis in LPS-treated mice and RANKL and LPS-induced OCs from BMMs. As shown in Figure 3, H&E and TRAP staining in histological sections of the femur in LPS treated mice showed that LPS treatment increased the number of TRAP positive OCs, and Mon treatment reduced the number of TRAP positive OCs in the bone tissue of LPS treated mice. Figure 4 also shows that Mon had no toxic effects on the viability of OCs, significantly decreased the number of TRAP+ multinucleated cells and also significantly reduced the size of OCs and the number of OC nuclei in a dose-dependent manner as evidenced by TRAP staining. In addition, Mon treatment in the early treatment period from day 1 to day 3 exerted stronger inhibitory effects on OCs than those from day 3 to day 6, suggesting that Mon inhibited the formation and differentiation of OCs mainly in the early phase of OC development.

### 3.4. Mon Attenuates TRAP Activity and Actin Ring Formation in BMMS

**Figure 5:**
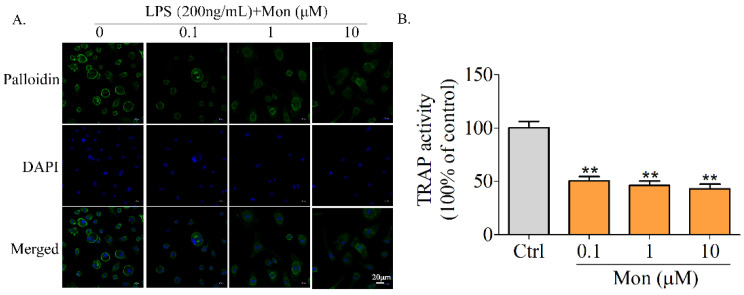
Mon attenuates TRAP activity and actin ring formation in BMMs. (**A**) Phalloidin staining to detect F-actin ring formation of osteoclast in response to Mon treatment. BMMs were incubated with M-CSF (30 ng/mL), RANKL (20 ng/mL) for 48 h and then treated with different concentrations of Mon (0.1, 1, and 10 µM) in present of 30 ng/mL M-CSF, 200 ng/mL LPS for 6 days and then stained with phalloidin (n = 3). (**B**) TRAP activity of osteoclasts in response to Mon treatment (n = 6). The data are expressed as means ± SD. ** *p* < 0.01 compared with ctrl group.

Knowing that TRAP activity is a key characteristic of OCs, we explored the effect of Mon on TRAP activity of OCs, and found that Mon at 0.1–10 µM significantly reduced the TRAP activity of OCs induced by RANKL and LPS (Figure 5B).

Knowing that actin ring formation following the rearrangement of the cytoskeleton is a characteristic of highly activated OCs, we subsequently investigated the effect of Mon on actin ring formation of OCs derived from BMMs. Complete and thick actin rings were clearly observed in OCs treated with LPS and RANKL, while actin rings in osteoclasts treated with Mon became thinner and their size and number were also decreased (Figure 5A), suggesting that Mon inhibited OS bone resorption by decreasing actin ring formation of OCs.

### 3.5. Mon Regulates Gene and Protein Expressions Involved in OC Formation and Differentiation

**Figure 6:**
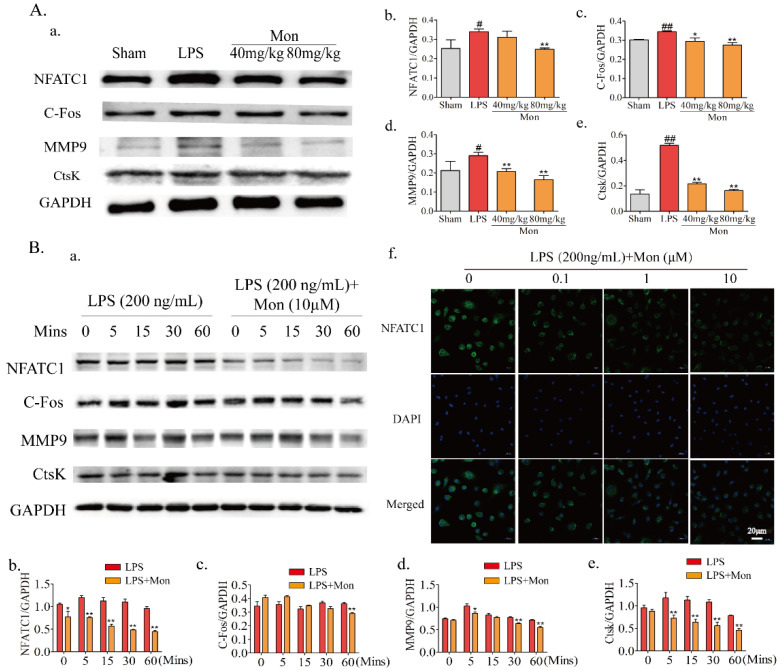
Mon regulates gene and protein expression involved into formation and differentiation of osteoclasts (n = 3). (**A**(**a**)) Representative imagines of Western blot for the expression of NFATc1, C-Fos, MMP9 and CtsK in bone tissue of LPS-stimulated model mice; (**A**(**b**–**e**)) quantitative analysis of NFATc1, C-Fos, MMP9, and CtsK, respectively, # *p* < 0.05, ## *p* < 0.01 compared with Sham group; * *p* < 0.05, ** *p*< 0.01 compared with LPS group. (**B**(**a**)) Representative imagines of Western blot for the expression of NFATc1, C-Fos, MMP9, and CtsK in osteoclast; (**B**(**b**–**e**)) quantitative analysis of NFATc1, C-Fos, MMP9, and CtsK, respectively, * *p* < 0.05, ** *p* < 0.01 compared with LPS group at corresponding time point. (**B**(**f**)) The translocation of NFATc1 of osteoclast stimulated by LPS. The data are expressed as means ± SD.

The formation and differentiation of OCs were modulated by transcription factors NFATc1 and C-Fos and their bone resorption activities were performed under the effects of MMP9 and CtsK. As shown in Figure 6, LPS treatment significantly enhanced the expression of OC transcription factors NFATc1 and C-Fos, and the expression of bone resorption proteins, such as MMP9 and CtsK in the bone tissue of LPS-treated mice, and Mon significantly inhibited the expression of NFATc1 and C-Fos, thereby inhibiting the expression of MMP9 and CtsK. Furthermore, Mon also suppressed the above transcription factors and key proteins and inhibited the nuclear translocation of NFATc1 in OCs from BMMs induced by LPS. These data indicate that Mon inhibited OC differentiation by attenuating the expression of bone resorption protein in LPS-induced inflammatory mice and OCs from BMMs.

### 3.6. Mon Inhibits Activation of the NF-κB Pathway in LPS-Treated Inflammatory Mice and OCs from BMMs

**Figure 7:**
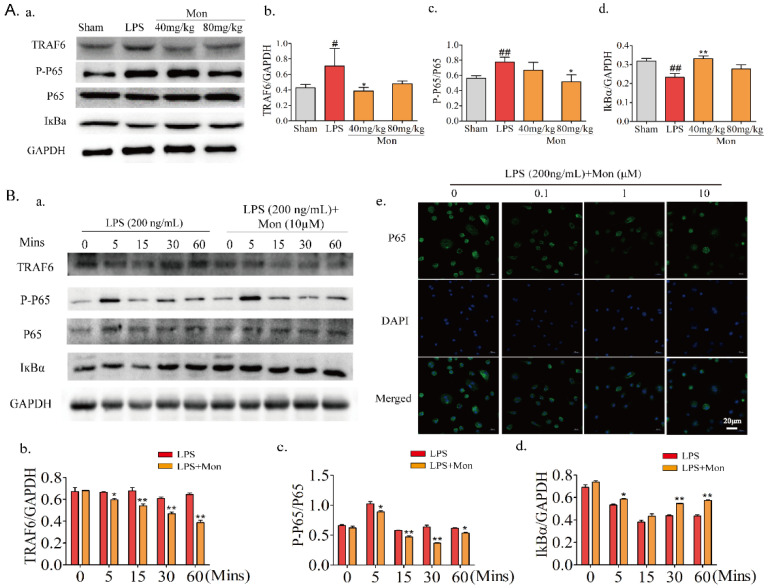
Mon inhibits the activation of NF-κB pathway in LPS-induced inflammatory mice and osteoclast derived from BMMs (n = 3). (**A**(**a**)) Representative imagines of Western blot for the expression of TRAF6, P-P65, P65, and IKBα in bone tissue of LPS-stimulated model mice; (**A**(**b**–**d**)) quantitative analysis of TRAF6, P-P65, P65, and IKBα, respectively. # *p* < 0.05, ## *p* < 0.01 compared with Sham group; * *p* < 0.05, ** *p*< 0.01 compared with LPS group. (**B**(**a**)) Representative imagines of Western blot for the expression of TRAF6, P-P65, P65, and IKBα in osteoclast; (**B**(**b**–**d**)) quantitative analysis of TRAF6, P-P65, P65, and IKBα in osteoclast, respectively, * *p* < 0.05, ** *p* < 0.01 compared with LPS group at corresponding time point. (**B**(**e**)) The translocation of P65 of osteoclast stimulated by LPS. The data are expressed as means ± SD.

As a dimeric transcription factor complex, NF-κB plays a critical role in osteoclastogenesis. As shown in Figure 7, Mon significantly inhibited the expression of TRAF6, thereby inhibiting the phosphorylation of P65 and the degradation of IKBα and inhibiting the activation of the NF-κB pathway in LPS-induced inflammatory mice and OCs from BMMs and also inhibited the nuclear translocation of P65 in OCs from BMMs.

### 3.7. Mon Suppresses Activation of the Akt/GSK-3β Pathway in LPS-Treated OCs from BMMs

**Figure 8:**
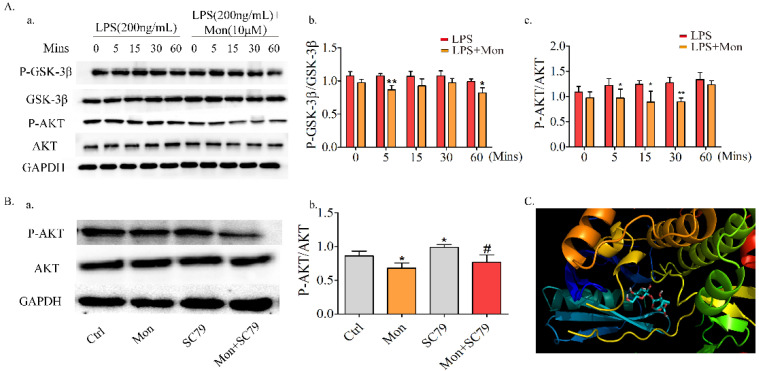
Mon suppresses the activation of Akt/GSK-3β pathway in LPS-treated osteoclast derived from BMMs and the molecular docking studies (n = 3). (**A**(**a**)) Representative imagines of Western blot for the expression of P-GSK-3β, GSK-3β, P-AKT and AKT in osteoclast; (**A**(**b**,**c**)) quantitative analysis of P-GSK-3β/GSK-3β and P-AKT/AKT, respectively. * *p* < 0.05, ** *p* < 0.01 compared with LPS group at corresponding time point. (**B**(**a**)) SC79, an Agonist of AKT reversed the regulatory effects of Mon on AKT pathway in osteoclast as showed by expression of key proteins in representative images of Western blot; (**B**(**b**)) quantitative analysis of P-AKT/AKT. * *p* < 0.05 vs. ctrl group, # *p* < 0.05 compared with SC79 group. (**C**) predicted binding mode of Mon with Akt. The data are expressed as means ± SD.

Akt/GSK-3β/NFATc1, which is the upstream molecule involved in the LPS-induced NF-κB pathway, is also involved in the regulation of OC formation and differentiation. As shown in Figure 8, Mon inhibited LPS-induced phosphorylation of Akt and GSK3β and the nuclear translocation of NFATC1 in OCs from BMMs, and these inhibitory effects of Mon were reversed by the AKT agonist SC79, indicating that Mon suppressed the activation of Akt/GSK-3β/NFATc1 in OCs.

### 3.8. Molecular Docking

To substantiate the above findings, we performed molecular docking analysis of Mon with AKT crystal structures (PDB ID: 6S9X) by adopting the Glidedock module from the Schrödinger suite. The docking estimation was performed by the docking score, knowing that a lower docking score value implies a better binding affinity between the protein and the ligand. The results showed that Mon with AKT had a good binding affinity with the docking score being −9.00 (Figure 8C).

## 4. Discussion

Inflammatory diseases such as SLE, RA, and CF often cause an increase in inflammatory cytokine levels in bone tissues and serum, leading to an increased OC activity, and subsequently bone loss and osteoporosis [4]. Mon is a natural compound obtained from some edible and medicinal plants and possess a definitely anti-inflammation activity [18]. It was found in our study that Mon inhibited bone loss and decreased the levels of inflammatory cytokines IL-6 and IL-β in LPS-induced bone loss mice, markedly reduced the number of LPS-stimulated TRAP (+) multinucleated cells in BMMs, and suppressed F-actin ring formation in LPS-induced OCs. These effects of Mon may be mediated by inhibiting NFATc1 via regulation of NF-κB and Akt/GSK3β signaling pathways.

LPS, an activator of inflammation, which can promote the secretion of pro-inflammatory cytokines, leading to a severe inflammatory response, is often used to induce inflammation in animal experiments [19]. Mon has been reported to possess anti-inflammatory effects as evidenced in various animal experiments, including mice of hot plate and writhing antinociceptive assays, acute paw edema induced by carrageenan in rats [20], the DSS-induced colitis model of rats [21], and secondary liver injury induced by chronic colitis [22]. In present study, Mon treatment significantly reduced the serum levels of IL-6 and IL-1β in LPS-induced inflammatory mice, further confirming that Mon significantly inhibited the inflammatory response in LPS-treated mice.

Accumulating evidence has demonstrated that inflammation impairs the OB function and stimulates the OC bone resorption activities [23]. LPS treatment can activate the immune cells, which in turn stimulates the inflammatory response and produces cytokines that increase the recruitment and activity of osteoclast, leading to bone loss [24]. It has been reported that injection of LPS for twice to mice within 10 days can cause acute inflammatory response and the activation of osteoclast and bone destruction [16]. Therefore, injection of LPS is currently one of the most recognized animal models for studying inflammatory bone loss [19,25] and has been widely applied in lots of investigation with the aim to observe the effects of drugs on inflammatory bone loss [26,27]. Consistent with the report in the literature [28], we found that LPS treatment increased levels of serum inflammatory factors and biochemical indicator of OC activities such as TRAP, RANK, and RANKL impaired the microarchitectures of the bone tissue, decreased the number of bone trabeculae, and promoted bone loss in the mice, while Mon treatment reversed these alterations in the bone tissue and serum biochemicals in LPS-induced inflammatory mice. The inflammatory cytokines, including TNF-a, IL-1β, and IL-6, directly or indirectly regulated OC formation and function. Mon treatment significantly reduced the serum levels of IL-6 and IL-1β in LPS-induced inflammatory mice, suggesting that Mon significantly inhibited the inflammatory response, OC activities and bone loss in LPS-induced mice.

LPS stimulation on OC formation is independent of RANKL induction [29]. Therefore, it is well recognized that searching for agents to reduce LPS-induced OC bone resorption is a promising strategy for the prevention of inflammatory bone loss [24]. In this study, we found that Mon, an iridoid glycoside existing in some edible and medicinal plants, significantly inhibited LPS-stimulated osteoclastogenesis in vitro at concentrations of 0.1–10 µM without causing significant toxicity. Mon also suppressed OC formation and differentiation in LPS-induced femur bone loss in vivo, which is consistent with our previous report that Mon inhibited LPS- and ovariectomy-induced bone loss in mice [30]. These results illustrated that Mon attenuated LPS-induced OC differentiation in vitro and bone destruction in vivo.

NFATc1 is a master transcription factor required for terminal differentiation of OCs [9,31]. It was reported that NFATc1 deficiency in primary OC precursors inhibited OC formation in response to RANKL or co-culture with OB in vitro [32]; NFATc1 regulated the expression of OC-specific genes, including TRAP, CTSK, DC-STAMP, and CTR and then stimulated OC formation and differentiation [28]. It was found in our study that LPS treatment increased the expression of NFATc1 in femurs of mice and OCs derived from BMMs, especially by enhancing nuclear translocation in OCs and the expression of their regulatory genes C-Fos, CtsK, and MMP9. Mon treatment inhibited the expression and nuclear translocation of NFATc1 and subsequently suppressed the expression of its target gene in LPS-induced mice and OCs, suggesting that inhibitory effect of Mon on OC formation and differentiation may be due to the suppression on NFATc1 activity, thus affecting F-actin ring formation and bone resorption.

The transcription and expression of NFATc1 are known to be regulated by NF-κB pathway [33]. NF-κB plays a key role in early signaling events by LPS-induced OC formation, and obstruction of its activation could lead to the inhibition of the OC bone resorptive function in vitro and in vivo [34]. In inflammation, RANKL, TNF-α, and IL-1 bind to RANK on the surface of OCs and subsequently recruit TRAF6, which in turn degrades IκBα, and then leads to the nuclear translocation of NF-κB P65, where it binds to the NFATc1 promoter, thus switching on the initial induction of NFATc1 [28]. In the present study, degradation of TRAF6 and IκBα and phosphorylation of P65 were increased in the bone tissue of LPS-induced mice and LPS-induced OCs, while Mon treatment reversed the stimulating effects of LPS on NF-κB pathway, indicating that Mon inhibited the activity of NFATc1, probably by suppressing activation of the NF-κB pathway.

The activities of NFATc1 also are modulated by Akt/GSK3β signaling pathway, which is critical for osteoclastogenesis [35]. In osteoclasts, the Akt signaling cascade is a key downstream pathway for three osteoclast surface receptors including c-fms, integrin ανβ3, and RANK. Akt is known to play a key role in osteoclast survival rather than in osteoclast differentiation through the phosphoinositide 3-kinase (PI3K) signaling pathway [36]. GSK3β is a central downstream effector of Akt signaling and its biological activity is inhibited by Akt-dependent phosphorylation, and a recent study showed the importance of the Akt-NFATc1 signaling axis in osteoclast differentiation. In addition, the study found that overexpression of Akt enhances formation of an inactive form of GSK3β (phospho-GSK3β) and nuclear localization of NFATc1 and that overexpression of a constitutively active form of GSK3β attenuates osteoclast formation through downregulation of NFATc1 [37]. GSK-3β inhibitors inhibited the formation and differentiation of LPS-induced mouse macrophages into OCs by inhibiting the expression of activated T-cell nuclear factor NFATc1 [37,38]. LPS stimulation increased the production of RANKL and M-CSF, and then activated Akt, which in turn phosphorylated GSK3β and inhibited its kinase activity [39]. This increased level of phospho-GSK3β (inactive form of GSK3β) could enhance the phosphorylation of NFATc1 and promote nuclear localization of NFATc1, resulting in osteoclastogenesis [10,35]. It was observed in our study that Mon suppressed the phosphorylation of Akt and GSK3β during OC differentiation, and the Akt agonist SC79 could partly reverse the effects of Mon, suggesting that the inhibitory effect of Mon on OC differentiation may be partly due to its inhibition on Akt/GSK3β signaling pathway.

Mon has various biological activities, such as anti-osteoarthritis, anti-apoptosis and anti-oxidation, and may prove to be a valuable therapeutic agent to treat bone loss-related diseases such as osteomyelitis, septic arthritis, and periodontitis [18]. Mon has been clinically used to treat RA in China recently. Patients with RA have elevated expression levels of both RANKL and inflammatory factors, including TNF-α. Mon has been reported to inhibit RANKL-induced osteoclastogenesis [40], and it may exhibit a potential therapeutic effect on bone erosion in RA by inhibiting both LPS- and RANKL-related signaling pathways to reduce the pathological over-activation of OCs. However, it is worth noting that Mon inhibited the osteoclastic activities at lower concentrations (0.1–10 µM), while Mon at higher doses (40 and 80 mg/kg) only can exert its pharmacological action, and this may be related with its lower bioavailability (2.04–3.69% and 8.29–16.12% in male and female rats, respectively) [41]. Therefore, further study should be focused on the structural optimization of Mon to improve its bioavailability.

## 5. Conclusions

**Figure 9:**
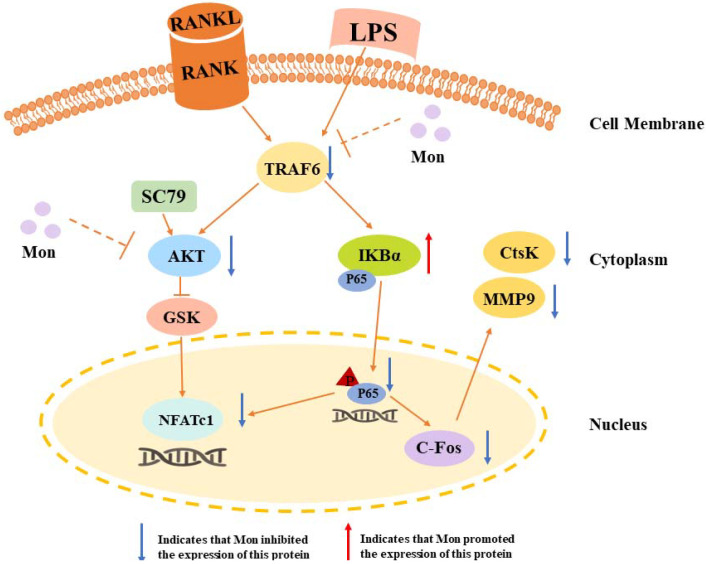
A schematic diagram illustrating the suppression of monotropein (Mon) on osteoclastogenesis via inhibiting NFATc1 through involving into NF-κB and AKT/GSK-3β pathway.

The results obtained in this study demonstrated that Mon prevented LPS-induced bone loss in vivo and suppressed LPS-induced osteoclastogenesis in vitro. In addition, Mon exhibited an inhibitory effect on LPS-induced osteoclastogenesis by inactivating NF-κB and AKT/GSK-3β-NFATc1 pathways (Figure 9). These findings provide a mechanistic insight into the application of Mon for LPS-mediated bone loss independent of RANKL. In spite of the novel findings in our study, the limitation of the LPS-induced bone loss model also should be addressed. Injection of LPS often induces acute inflammatory response. Hence, it was justifiable that this model was used to explore the mechanism of Mon on inflammatory bone loss, but it is not enough to definitely verify the effectiveness of Mon on bone loss induced by chronic inflammation. Further research needs to be conducted on model animals of inflammatory diseases, such as SLE, RA, and CF, so as to provide perspectives for the application of Mon and edible and medicinal plants containing this compound in inflammatory diseases.

## Data Availability

Not applicable.
